# Our Advertising Friends

**Published:** 1871-01

**Authors:** 


### OUR ADVERTISING FRIENDS.

For the information of our readers we will state that, one object of our Journal is to bring before them the names of first-class business-houses, who are legitimately allied with the profession.

Our conception of the mission of a Medical Journal, is not simply to confine its pages to the discussion of questions in the various departments of medical science, but that a larger, broader field should be occupied. In other words, it is the province of the medical journalist to bring before the professional reader everything that shall tend to promote his efficiency in the "Science and the Art" of his calling. Hence it is our duty, as far as possible, to make him familiar with reliable Druggists, Manufacturers of Pure, Standard Extracts, Manufacturers of Pure Chemicals—as his success, as a practitioner, depends so much upon the purity and reliability of drugs and chemicals. Also to introduce to his notice the newest and most approved instruments necessary to the accomplishment of skillful and successful operations; or those facilitating diagnosis or the application of remedial agents. With instrument makers of character; with publishing houses, offering new standard publications in medical and surgical science—and whatever else may serve to add efficiency to professional skill, or open up new avenues of thought to aid him in the struggle between science and disease.

We therefore ask our readers to give our advertising columns that attention they should command—not only in this number of the Companion, but in those that are to follow. We feel assured that we shall be instrumental, from time to time, in presenting in our columns, many things of the utmost value to them in the prosecution of their professional duties.

